# A Novel Podophage StenR_269 Suggests a New Family in the Class Caudoviricetes

**DOI:** 10.3390/v15122437

**Published:** 2023-12-15

**Authors:** Vyacheslav I. Yakubovskij, Vera V. Morozova, Yuliya N. Kozlova, Artem Y. Tikunov, Igor V. Babkin, Alevtina V. Bardasheva, Elena V. Zhirakovskaya, Ivan K. Baykov, Galina B. Kaverina, Nina V. Tikunova

**Affiliations:** 1 Laboratory of Molecular Microbiology, Institute of Chemical Biology and Fundamental Medicine Siberian Branch of Russian Academy of Sciences, Novosibirsk 630090, Russia; yakubovskij97@gmail.com (V.I.Y.); arttik1986@gmail.com (A.Y.T.); i_babkin@mail.ru (I.V.B.); ivan_baykov@mail.ru (I.K.B.);; 2 Faculty of Natural Sciences, Novosibirsk State University, Novosibirsk 630090, Russia

**Keywords:** *Stenotrophomonas rhizophila*, phage, podovirus morphotype, *Zobellviridae*, comparative genomics, Caudoviricetes, N4-like capsid protein

## Abstract

*Stenotrophomonas rhizophila* was first discovered in soil; it is associated with the rhizosphere and capable of both protecting roots and stimulating plant growth. Therefore, it has a great potential to be used in biocontrol. The study of *S. rhizophila* phages is important for a further evaluation of their effect on the fitness and properties of host bacteria. A novel phage StenR_269 and its bacterial host *S. rhizophila* were isolated from a soil sample in the remediation area of a coal mine. Electron microscopy revealed a large capsid (~Ø80 nm) connected with a short tail, which corresponds to the podovirus morphotype. The length of the genomic sequence of the StenR_269 was 66,322 bp and it contained 103 putative genes; 40 of them encoded proteins with predicted functions, 3 corresponded to tRNAs, and the remaining 60 were identified as hypothetical ones. Comparative analysis indicated that the StenR_269 phage had a similar genome organization to that of the unclassified *Xanthomonas* phage DES1, despite their low protein similarity. In addition, the signature proteins of StenR_269 and DES1 had low similarity and these proteins clustered far from the corresponding proteins of classified phages. Thus, the StenR_269 genome is orphan and the analyzed data suggest a new family in the class Caudoviricetes.

## 1. Introduction

The genus *Stenotrophomonas* belongs to the family *Xanthomonadaceae*. The members of this genus are ubiquitous and can be isolated from various habitats, including the rhizo-sphere, soil, and plant surfaces. The reason for such wide distribution is their ability to utilize various carbon sources and their resistance to heavy metals. *Stenotrophomonas* spp. metabolize phenols and xenobiotics and some of them are able to stimulate plant growth [1].

*Stenotrophomonas rhizophila* was first described in 2002 as a rhizobacterium associated with plants and possessing anti-fungal activity [2]. It has been reported that *S. rhizophila* strains promote root growth and can be used as biocontrol agents [3,4]. Like many other members of the genus, *S. rhizophila* has high metabolic activity and is able to digest various aromatic compounds, such as phenol, catechol, cresols, benzoic acid, vanillic acid, hydroquinone, and polycyclic pharmaceutical naproxen [1]. In addition, *S. rhizophila* is involved in the decomposition of lignocellulose together with some other soil bacteria [5]. *S. rhizophila* has a high biocontrol potential that is mainly associated with the production of keratinases, proteinases and chitinases [6]. Moreover, it has been shown that the plant-associated strain *S. rhizophila* DSM14405T (biocontrol agent) possesses unique genes for the synthesis and transport of the plant-protective spermidine, plant cell wall-degrading enzymes, and high salinity tolerance [7,8]. These peculiarities make *S. rhizophila* a perspective candidate for applications in agriculture and for soil bioremediation [3,4]. Bacteria used as biocontrol agents in agriculture are likely to encounter environmental phages, and those can affect bacterial fitness and characteristics [9]. Thus, studying *S. rhizophila* phages is useful for further understanding their potential impact on the bacterial host.

To date, 65 complete genomes of *Stenotrophomonas* phages are available in the GenBank database (https://www.ncbi.nlm.nih.gov/nucleotide, accessed on 10 November 2023) (Data S1). These include 60 phages that belong to the class Caudoviricetes (tailed phages with linear double-stranded DNA), and five phages that belong to the *Inoviridae* family (filamentous phages with circular single-stranded DNA). *Stenotrophomonas* caudate phages are diverse in genome content, life cycle and capsid morphotype [10,11,12,13,14,15,16,17]; 26 of them were distributed into six different families, namely *Autographiviridae* (*n* = 11), *Mesyanzhinoviridae* (*n* = 6), *Schitoviridae* (*n* = 5), *Straboviridae* (*n* = 2), *Casjensviridae* (*n* = 1) and *Peduoviridae* (*n* = 1). The remaining 34 tailed phages were assigned to the subfamily *Beaumontvirinae* (*n* = 6) and to the genera *Menderavirus* (*n* = 7), *Delepquintavirus* (*n* = 3) and *Septimatrevirus* (*n* = 2); the last 16 *Stenotrophomonas* bacteriophages are designated as unclassified representatives of the class Caudoviricetes (https://www.ncbi.nlm.nih.gov/nucleotide, accessed on 10 November 2023). In total, 22 phages have the podovirus morphotype; these include members of the families *Autographivirinae* and *Schitoviridae,* and four unclassified members of the class Caudoviricetes. According to GenBank data, 61 phages reproduce on *Stenotrophomonas maltophilia* as a host, and only 4 of them infect the *Stenotrophomonas* sp. (https://www.ncbi.nlm.nih.gov/nucleotide, accessed on 10 November 2023) (Supplementary Data S1). Phages specific to *S. rhizophila* have not been detected.

In this study, a novel phage, StenR_269, and its bacterial host, *S. rhizophila*, were isolated. Electron microscopy revealed a large capsid (~Ø80 nm) connected with a short tail; thus, the phage had a podovirus morphotype. The length of the StenR_269 genome sequence was 66,322 bp, and a comparative analysis of the genome revealed only a low level of similarity (~5%) to the unclassified *Xanthomonas* phage DES1. The most similar classified phages were members of *Zobellviridae* family with a level of similarity <1%; therefore, the genome of the phage StenR_269 is unique. The obtained data indicate the further creation of a new family in the class Caudoviricetes.

## 2. Materials and Methods

### 2.1. Bacterial Host Strain Isolation and Identification

An environmental isolate of *S. rhizophila* was found in a soil sample from the remediation area of a coal mine in Kemerovo region, Siberia, Russia. Bacterium isolation was carried out as described previously [18]. Briefly, 2 g of soil was suspended in 10 mL of sterile phosphate-buffered saline, PBS, at pH 7.5. Then, suspension was clarified using low-speed centrifugation (2000× *g*, 5 min), and aliquots of 10-fold dilutions of the suspension were poured onto plates containing Nutrient agar (Microgen, Obolensk, Russia). Plates were incubated overnight at 25 °C and the resulting various colonies were independently passaged three or more times on the plates containing the Nutrient agar to obtain pure cultures. Identification of bacterial species was performed via sequencing a fragment of the 16S rRNA gene with a length of 1308 bp. Primers 16s-8-f-B 5′-AGRGTTTGATCCTGGCTCA-3′ and 16s-1350-r-B 5′-GACGGGGCGGTGTGTACAAG-3′ were used for amplification and sequencing as described previously [19]. The 16S rRNA sequence was submitted to the NCBI GenBank database (ID OP393922). The strain *S. rhizophila* CEMTC 4565 was deposited in the Collection of Extremophilic Microorganisms and Type Cultures (CEMTC) of the Institute of Chemical Biology and Fundamental Medicine SB RAS, Novosibirsk, Russia.

### 2.2. Phage Isolation and Propagation

Phage StenR_269 was isolated from the same soil sample that was used for obtaining host strain *S. rhizophila* CEMTC 4565. Phage isolation was carried out as described previously [18] with minor changes. Briefly, 0.5 g of soil was suspended in 10 mL of sterile phosphate-buffered saline, PBS, at pH 7.5. Then, the soil–water suspension was clarified via centrifugation at 10,000× *g* for 15 min and sterilized via filtration through a 0.22 μm filter (Wuxi Nest Biotechnology, Wuxi, China). Screening for the presence of phages was carried out by dropping 10 μL aliquots of filtrate onto a fresh lawn of *S. rhizophila* CEMTC 4565 in the top agar (Condalab, Madrid, Spain). Plates were incubated overnight at 25 °C and then examined for the presence of single-phage plaques. The obtained single-phage plaque was cut from the top agar, suspended in sterile PBS, and incubated with stirring overnight to extract phage particles from the agar. The next day, ten-fold dilutions of phage-containing eluate were added to the fresh layer of *S. rhizophila* CEMTC 4565 in the top agar. The plates were incubated overnight at 25 °C to obtain individual phage plaques. The resulting individual plaques were examined for the identity of their morphology and used for the subsequent isolation of pure phage culture.

To amplify the phage StenR_269, 50 mL of exponentially growing *S. rhizophila* CEMTC 4565 in the Nutrient Broth, NB (Condalab, Madrid, Spain) was infected with the phage, and the multiplicity of infection (MOI) was 0.1. The infected bacterial culture was incubated with shaking at 25 °C for some hours. Afterwards, the obtained phage particles were purified and concentrated as described previously [20]. Briefly, bacteria and debris were centrifuged at 10,000× *g* for 30 min and phages were precipitated from the supernatant using polyethylene glycol 6000 (AppliChem, Darmstadt, Germany). Phage-containing precipitate was dissolved in 500 µL of STM buffer (0.59 g of NaCl; 7.88 g of Tris-HCl, pH 7.5; and 2.38 g of MgCl_2_ per 1 L).

### 2.3. Phage Particle Morphology, Host Range Assay and Biological Characteristics

The morphology of phage particles was studied using transmission electron microscopy with preliminary negative staining as described previously [18]. Biological characteristics of the phage StenR_269 were evaluated using *S. rhizophila* CEMTC 4565 as a host. All experiments were performed twice, each in three technical repeats. Graphs were constructed and statistical analysis was performed using GraphPad Prizm v. 8.0.1.

Phage adsorption experiments were performed as described previously [21]. Briefly, the phage StenR_269 was added to exponentially growing *S. rhizophila* CEMTC 4565 with an MOI of 0.001. The phage–bacteria incubation mixture was shaken at 25 °C for 30 min and aliquots were taken every minute to determine the titer of free phages. The adsorption rate constant was calculated in accordance with Kropinsky [21]. 

One-step growth and burst size experiments were carried out as described previously [22] with slight modifications. In total, 10 mL of an exponentially growing *S. rhizophila* CEMTC 4565 was centrifuged at 8000× *g* for 10 min, and then the pellet was resuspended in 0.5 mL of NB (Condalab, Spain). Phage StenR_269 with an MOI of 0.001 was added to the cell suspension, and the mixture was incubated for 5 min at 25 °C for phage adsorption. Then, the cells were pelleted via centrifugation and resuspended in 10 mL of NB. Incubation was continued for 1 h at 25 °C. Culture aliquots were collected every 2 min, immediately diluted and plated for phage titration. The burst size was calculated by dividing the total number of phage progenies produced during a single round of infection by the total number of infected cells. 

The lytic activity of the StenR_269 phage was evaluated as described earlier [23] with our modifications. An exponentially growing culture of *S. rhizophila* CEMTC 4565 (20 mL with the titer 5 × 10^7^ cfu/mL) was poured equally into two tubes. The first portion (10 mL) of the culture was mixed with the phage StenR_269 at an MOI of 0.1; the second was grown further without the phage. Then, both the phage-free bacterial culture and the phage–bacteria mixture were incubated while shaking at 25 °C for 19 h. Aliquots were taken from both cultures once an hour for the first nine hours, and the last aliquots were taken the next morning after 19 h of incubation. Aliquots were immediately diluted in NB, then appropriate dilutions were spread on Nutrient agar plates and incubated overnight at 25 °C. The next day, colonies were counted and bacterial titers were determined for each point. The obtained data were used to calculate a multistep bacterial reduction curve for the phage StenR_269. The host range for the phage StenR_269 was determined using the spot assay method, as described previously [24]. A total of 64 strains of *Stenotrophomonas* spp. from the CEMTC ICBFM SB RAS were tested that included clinical and environmental strains, and strains isolated from insects (Supplementary Data S2).

### 2.4. Genome DNA Purification and Complete Genome Sequencing

Genome DNA purification was carried out as described previously [25]. Briefly, DNase and RNase (Thermo Fisher Scientific, Waltham, MA, USA) were added to the phage suspension, each to a final concentration of 5 μg/mL, and the mixture was incubated at 37 °C for 30 min. Next, the phage suspension was supplemented with a 1/25 volume of 0.5 M EDTA (pH 8.0), 1/20 volume of 10% SDS solution, and proteinase K (Thermo Fisher Scientific, Waltham, MA, USA) to a final concentration of 100–200 μg/mL. Then, the suspension was incubated at 55 °C for 3 h and phage DNA was purified via phenol/chloroform extraction. Further, ethanol supplemented with a 1/30 volume of 3 M sodium acetate (pH 4.8) was added to precipitate the DNA. Covaris Ultrasonicator (Covaris, Moburn, MA, USA) was applied for phage DNA fragmentation. NEB Next Ultra II DNA Library Prep Kit for Illumina and NEB Next multiplex oligos for Illumina (both from New England BioLab, Ipswich, MA, USA) were used for DNA library construction. Sequencing was performed using MiSeq Benchtop Sequencer and the MiSeq v.2 reagent kit (2 × 250 base reads) (Illumina Inc., San Diego, CA, USA). Quality control of the obtained data and the removal of adapter sequences were performed using the Trimmomatic tool [26]. The phage genome was assembled de novo using SPAdes genome assembler v.3.15.2 [27] (http://cab.spbu.ru/software/spades, accessed on 2 July 2023). The sequencing coverage was estimated to be 405.

### 2.5. Analysis of Phage Genome

An initial annotation of the StenR_269 genome was carried out using Rapid Automated Annotation Service (RAST) v.2.0 [28] (https://rast.nmpdr.org, accessed on 10 October 2023). Then, the obtained annotation was verified manually with a BLASTX and BLASTP search for sequences deposited in the NCBI GenBank database (https://ncbi.nlm.nih.gov, accessed on 23 October 2023). In addition, proposed protein functions were verified using InterProScan and HHpred tools [29,30]. The presence of tRNA genes was detected using tRNAscan-SE [31]. PhageTerm tool v.1.0.12 [32] was applied to determine phage genome termini and the DNA packaging strategy. Signal sequences in proteins were detected using SignalP 6.0 [33]. The genome of StenR_269 was deposited to the NCBI GenBank database with the accession number OR838459. The annotated genome of the StenR_269 phage was added to Supplementary Data S3. To estimate the taxonomy of the phage StenR_269, a comparative proteomic phylogenetic analysis was performed on Viral Proteome Tree Server (ViPTree) [34] (https://www.genome.jp/viptree, accessed on 1 November 2023). In addition, the VIRIDIC tool [35] (https://rhea.icbm.uni-oldenburg.de/viridic, accessed on 6 November 2023) was used to calculate the similarity of the studied genome to other phage genomes.

### 2.6. Phylogenetic Analysis of Phage Proteins

Phage proteins were compared with non-redundant protein sequences (nr) and reference proteins (refseq_protein) of the NCBI GenBank database using BLASTP (https://blast.ncbi.nlm.nih.gov/, accessed on 28 October 2023). Proteins of interest were extracted for further analysis. Protein sequences were aligned and phylogenetic analysis was performed in the MEGA 7.0.21 software [36].

## 3. Results

### 3.1. StenR_269 Phage Particle Morphology, Host Range Assay and Biological Characteristics

**Figure 1:**
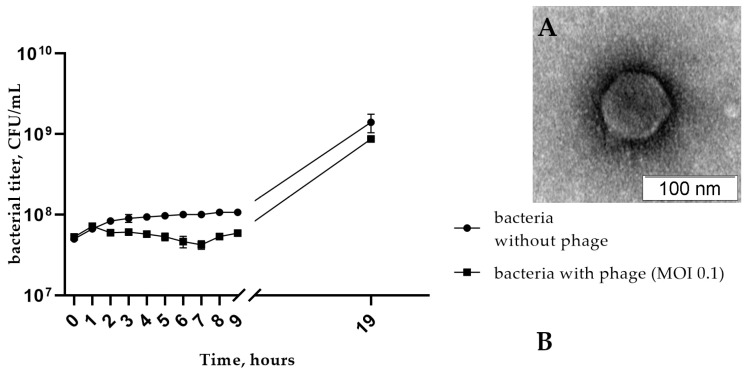
(**A**) Electron micrograph of StenR_269 phage particles negatively stained with 1% uranyl acetate; (**B**) the multistep bacterial reduction curve for *S. rhizophila* CEMTC 4565 in the life cycle of the phage StenR_269.

The phage StenR_269 formed small turbid plaques with a diameter of about 0.5 mm on the lawn of the host strain. Electron microscopy revealed an icosahedral head (Ø ~80 nm) connected to a short non-contractile tail (Figure 1A). Thus, the morphology of the phage StenR_269 particles corresponded to that of the morphotype of podovirus.

More than 65% of StenR_269 particles attached to host cells within 12 min in phage adsorption experiments. The adsorption rate constant of the phage StenR_269 for *S. rhizophila* CEMTC 4565 cells was calculated as 1.6 × 10^−8^ mL/min.

A one-step growth assay for StenR_269 revealed a latent period of about 35 min with a burst size of ~45 phage particles per infected cell. The multistep bacterial reduction experiments showed that phage reduced the titer of bacteria by half of the order compared with the control culture (7 h from the beginning of the experiment; Figure 1B). Consequently, the phage does not have high lytic activity to its host under these growth conditions.

The host range of the phage StenR_269 was analyzed using 64 strains of *Stenotrophomonas* spp., which included strains of *S. rhizophila* (*n* = 14), *S. maltophilia* (*n* = 21), *Stenotrophomonas lactitubi* (*n* = 3), *Stenotrophomonas chelatiphaga* (*n* = 5), *Stenotrophomonas pavanii* (*n* = 2), *Stenotrophomonas geniculata* (*n* = 3), *Stenotrophomonas bentonitica* (*n* = 1), *Stenotrophomonas tumulicola* (*n* = 1), *Stenotrophomonas acidaminiphila* (*n* = 1) and *Stenotrophomonas* sp. (*n* = 13) (Data S2). It was found that the phage StenR_269 has a narrow host range and infects only the bacterial host *S. rhizophila* CEMTC 4565 from 64 tested strains. 

### 3.2. Genome Characteristics

**Figure 2:**
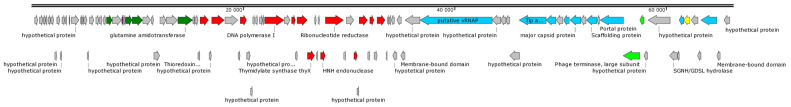
StenR_269 genome map. DNA metabolism genes are marked with red arrows; putative structural genes are marked with blue arrows; subunits of terminase are marked with light-green arrows; lysozyme marked with yellow arrows; genes corresponding to glutamine synthesis marked with dark-green arrows; other genes are marked with gray arrows.

The length of the genome of the phage was 66,322 bp with a GC content of 49.73%, which is far from the ~67.5% GC content in *S. rhizophila* genomes (reference strain DSM 14405; RefSeq: GCF_000661955.1). The termini of the phage genome were determined using the PhageTerm tool (Data S4). In total, 104 putative open reading frames (ORFs) were identified using the RAST tool, and 43 of them encoded products with predicted functions (40 proteins and 3 tRNA), while the remaining 61 ORFs were defined as hypothetical ones. Three tRNA genes were located at the beginning of the genome and they were identified as the glutamine (TTG), glycine (TCC) and arginine (TCT) tRNA genes. ORFs can be divided into two clusters according to their orientation in the StenR_269 genome; however, the exact definition of genetic clusters is difficult due to the presence of genes encoding hypothetical proteins (Figure 2).

A group of hypothetical genes are located at the beginning of the genome. Presumably, this group contains the early genes that are responsible for switching the bacterial host cell metabolism to the synthesis of phage RNA and phage proteins. This group is followed by the genes that are associated with glutamine synthesis. Since the genomes of the phage and the host bacterium differ significantly in GC-content, the presence of the glutamine synthesis genes and the tRNA-Glu gene probably improves the fitness of the phage. 

Next to the glutamine synthesis genes, the genome contains genes encoding enzymes for DNA replication, namely DNA polymerase, DNA primase and DNA helicase. The last cluster is located on the opposite strand of DNA and contains genes responsible for capsid synthesis, phage DNA packaging and cell lysis. Phage endolysin was classified as a lysozyme and a signal peptide was not found in its sequence using the SignalP 6.0 tool (Data S5). Note that major capsid protein (gene position 50,930–51,937 bp) was attributed to N4-gp56 family proteins. 

A large hypothetical protein (2270 *aa*) was found (gene position 36,735–43,547 bp) in the cluster of late genes of the StenR_269 genome. In order to determine the function of this protein, HHpred and InterProscan tools were used, but this did not allow us to reliably determine the function of this protein. Analysis using PSIPRED 4 (http://bioinf.cs.ucl.ac.uk/psipred, accessed on 17 November 2023) showed that the secondary structure of the protein predominantly contains alpha helices and coiled regions. Modeling of protein fragments using Alpha Fold 2 (https://colab.research.google.com/github/sokrypton/ColabFold/blob/main/AlphaFold2.ipynb, accessed on 19 November 2023) led to models with low confidence, also mainly consisting of alpha helices and coiled regions. In addition, this protein does not contain cysteine residues. All these characteristics (the large size, absence of cysteine residues, alpha-helical secondary structure) allow us to assume that this protein could be a single-subunit virion-associated RNA polymerase (vRNAP), like the RNA polymerase of the N4 phage [37].

### 3.3. Comparative Analysis of the StenR_269 Genome and Its Taxonomy

**Figure 3:**
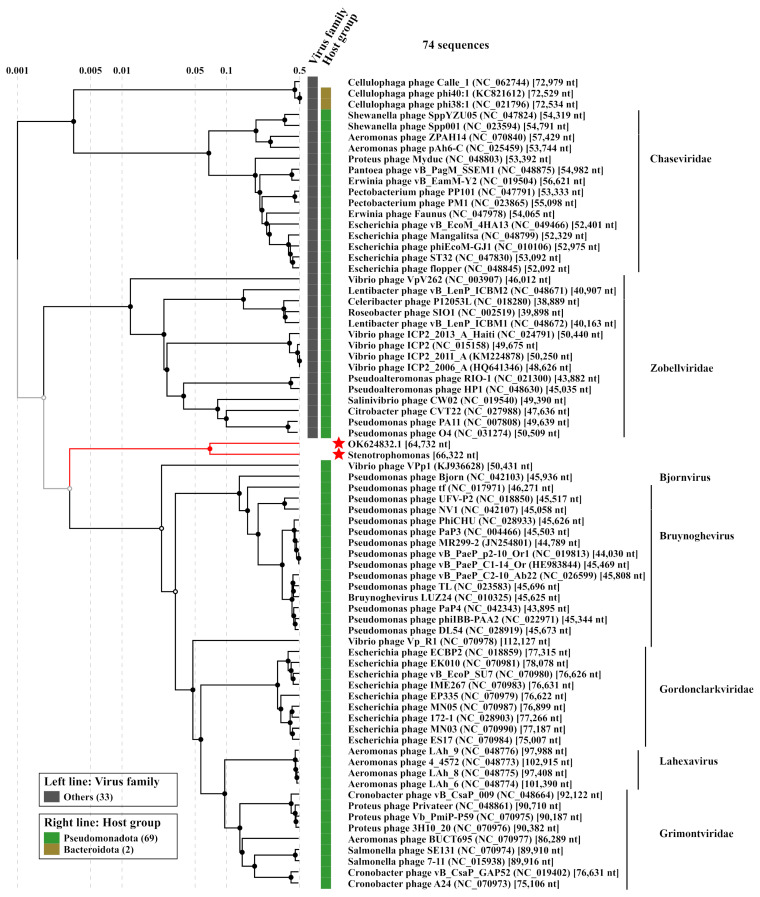
ViPTree analysis of the phage StenR_269. The studied genome of StenR_269 and the most similar genome of the *Xanthomonas* phage DES1 (OK624832) are marked with red asterisks.

**Figure 4:**
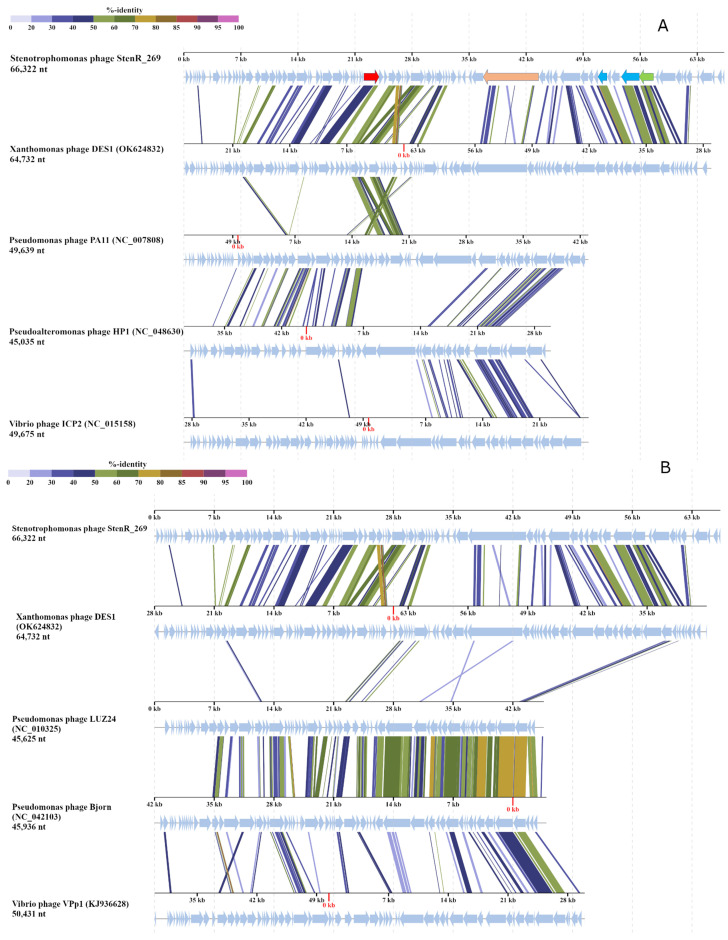
StenR_269 and DES1 genome alignment prepared using ViPTree tool. (**A**) Comparison with *Zobellviridae* phages; several genes of the StenR_269 genome are marked. Gene encoding DNA polymerase is marked in red, putative vRNAP is marked in beige, capsid and portal proteins are light blue, and the large subunit of terminase is light green. (**B**) Comparison with *Pseudomonas* phage Bjorn (*Bjornvirus* genus), *Pseudomonas* phage LUZ24 (*Bruynoghervirus* genus) and *Vibrio* phage VPp1 (unclassified Caudoviricetes).

To evaluate the taxonomy of the phage StenR_269, its genome was compared with available phage sequences from the NCBI GenBank database via a BLASTN search (accessed on 21 October 2023). A similarity of more than 90% was found with incomplete genomes of phage spp. isolates 227 (MN855844; sequence length: 9692 bp) and 386 (MN855896; sequence length: 6481 bp) (Supplementary Data S6). These sequences were found in the insect metagenome obtained from *Apis mellifera* [38]. No other phage sequences similar to that of the StenR_269 genome were found through the BLASTN search. The BLASTP and BLASTX search revealed a limited similarity of the StenR_269 proteins with those of the Xanthomonas phage DES1 (OK624832, unclassified Caudoviricetes), Citrobacter phage CVT22 (NC_027988, *Zobellviridae*), *Vibrio* phage VpV262 (NC_003907, *Zobellviridae*) and some unclassified phages. Then, ViPTree analysis was performed (Figure 3) and it was revealed that the phages StenR_269 and DES1 form a cluster that is genetically distant from other phages available in the Virus–Host database (https://www.genome.jp/virushostdb (accessed on 21 October 2023)). The VIRIDIC tool was used to estimate genome similarity and it was found to be very low (Data S7). Despite the low level of similarity between the StenR_269 and DES1 proteomes, ViPTree alignment revealed a clear similarity in the organization of the StenR_269 and DES1 genomes (Figure 4). Both genomes contained two clusters of genes that were grouped according to their function and orientation in the genome. 

A comparison of the StenR_269 and DES1 phages with other classified phages indicated that the genome organization of members of *Zobellviridae*, *Bjornvirus* and *Bruynoghevirus* is somewhat similar to that of those of StenR_269 and DES1 including the same orientation of genome clusters (Figure 4). However, the StenR_269 and DES1 genomes were at least 14 kb longer than the genomes of the classified phages. In addition, relatively large ORFs were located in the analyzed *Zobellviridae* genomes at positions corresponding to the positions for the vRNAP-encoding ORFs in the StenR_269 and DES1 genomes (Figure 4). The proteins that are encoded by the *Zobellviridae* genomes were previously annotated as hypothetical or internal virion proteins (protein ID UFK09611 for the phage DES1, YP_001294628 for the phage PA11, YP_009966391 for the phage HP1 and YP_004251177 for the phage ICP2). Like the putative vRNAP of StenR_269, the proteins from the *Zobellviridae* phages did not include cysteine residues and their secondary structure contained alpha helices and coiled regions.

### 3.4. Phylogenetic Analysis of Essential Phage Proteins

**Figure 5:**
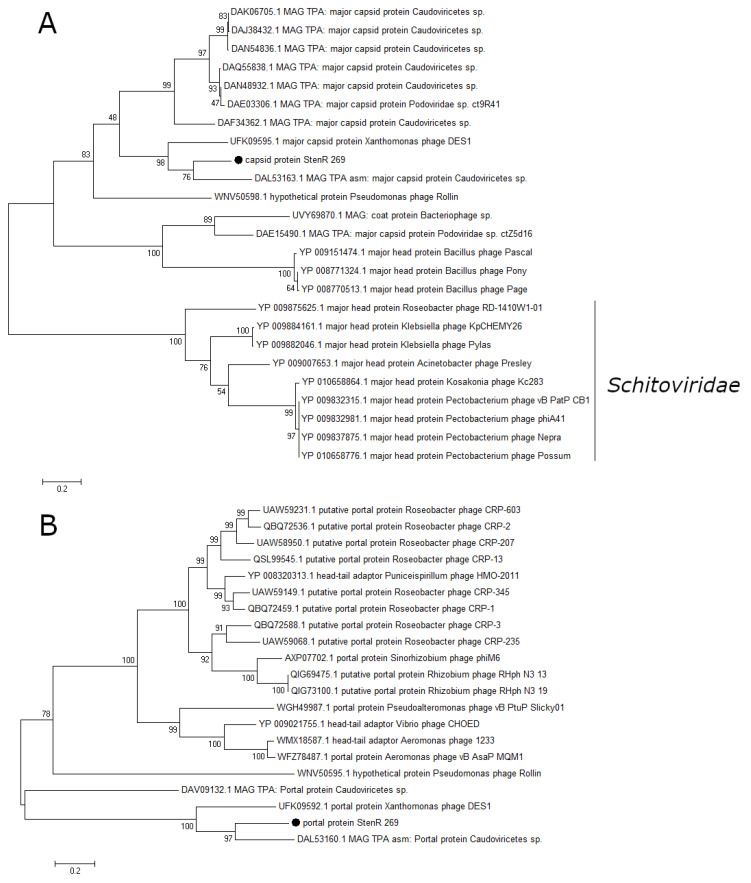
Phylogenetic analysis of structural proteins of the phage StenR_269. Capsid protein (**A**) and portal protein (**B**). Sequences were aligned using CLUSTALW, and the phylogenetic tree was constructed using the MEGA 7.0.21; maximum likelihood method with a bootstrap value of 1000 being applied.

**Figure 6:**
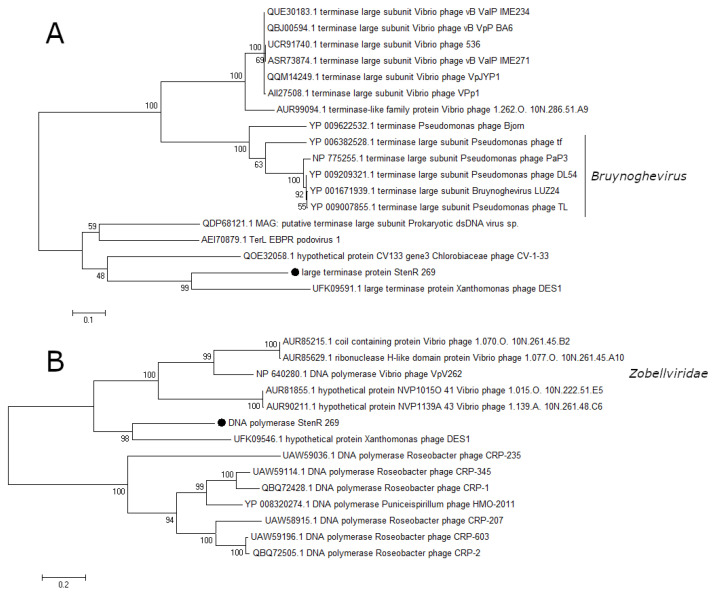
Phylogenetic analysis of the essential proteins of the phage StenR_269; terminase large subunit (**A**) and DNA polymerase (**B**). Sequences were aligned using CLUSTALW, and the phylogenetic tree was constructed using the MEGA 7.0.21; maximum likelihood method with a bootstrap value of 1000 being applied.

A number of essential phage proteins of the phage StenR_269, namely capsid protein, portal protein, large terminase subunit, and DNA polymerase were analyzed. The results of phylogenetic analysis confirmed the limited similarity between the phages StenR_269 and DES1 and their distant position from other classified phages. It was revealed that the StenR_269 virion proteins, namely the capsid and portal protein, showed limited similarity to the proteins of the N4-like and unclassified phages, respectively (Figure 5). In addition, the terminase large subunit and DNA polymerase have similarities with the proteins of phages of the genus *Bruynoghevirus* and the family *Zobelleviridae*, respectively (Figure 6). Thus, both StenR_269 and DES1 phages can be representatives of different genera and later of a novel phage family.

## 4. Discussion

In this study, the phage StenR_269, which is specific to *Stenotrophomonas rhizophila*, was described for the first time. According to the electron microscopy study, the StenR_269 possesses the morphotype of podovirus with a relatively large capsid (Ø ~80 nm), which suggests a large genome size. It was found that the genome of StenR_269 was more than 66 kbp in size and its sequence showed low similarity to other available sequences in the GenBank database. Only one phage, unclassified *Xanthomonas* phage DES1, had some genome similarity (~5%) to StenR_269. However, a similar organization of genomes was found in both phages, since two large genetic clusters oriented in opposite directions were found in both genomes. The first cluster contained putative early genes, genes encoding glutamic amino acid synthesis and genes corresponding to virus replication. The second cluster contained genes responsible for capsid assembly, DNA packaging and bacterial lysis. Notably, two phage genome fragments (9692 bp and 6481 bp) that showed high similarity (>90%) to StenR_269 were found in the metagenome of *Apis mellifera* [38]. This fact indicates that StenR_269 can be the first representative of a new genus and that other members of this genus can be found in the metagenomes of herbivorous insects. 

Among the classified phages, members of the family *Zobellviridae* and the genus *Bruynoghevirus* had at least some similarity, while the level of genome similarity was <1%. Nevertheless, these classified phages had similar genome organization, although their genomes were less than 50 kb. The phylogenetic analysis of signature proteins confirmed the distant taxonomic position of the StenR_269 and DES1 phages, since no close protein sequences were found among the classified phages. Notably, similar protein sequences were found only in metagenomic data. Thus, the StenR_269 and DES1 phages differ significantly from other known phages and the data obtained suggest a new family in the Caudoviricetes class. 

According to the analysis of biological characteristics, the studied phage was not highly lytic towards its host; it only slightly reduced the growth of bacteria. It is likely that most of host cells could survive and grow in the bacteria–phage mixture. The mechanism of this phenomenon is currently unclear, which may be due to the absence of phage receptors on the cells, a number of bacterial immunity factors, pseudolysogeny and some other reasons. Although integrases have not been clearly detected in the StenR_269 genome, the latter contains many hypothetical genes that could potentially influence the fitness and characteristics of host bacteria, including their ability to produce biofilm and survive in the rhizosphere. The possible effect of the phage on a bacterium can be evaluated by sequencing the genome of the host bacterium, as well as by studying metabolic activity (gene expression) of the host bacterium in the absence of and under the influence of the phage.

## Data Availability

The genome sequence of the *Stenotrophomonas* phage StenR_269 and 16S rRNA sequences of *Stenotrophomonas* strains are available in the GenBank database.

## Supplementary Materials

The following supporting information can be downloaded at https://www.mdpi.com/article/10.3390/v15122437/s1. Supplementary Data S1. List of *Stenotrophomonas* phages available in Genbank database; Supplementary Data S2. List of *Stenotrophomonas* spp. used for host range determination; Supplementary Data S3. The StenR_269 genome annotation; Supplementary Data S4. Phage term analysis; Supplementary Data S5. Analysis of the protein sequence of the StenR_269 endolysin using SignalP 6.0 tool; Supplementary Data S6. Comparative analysis of the StenR_269 genome with the phage isolates 227 (MN855844) and 386 (MN855896) extracted from the metagenomic data; Supplementary Data S7. StenR_269 genome similarity analysis performed using VIRIDIC tool.
