# Supplementary material for: A Novel Podophage StenR_269 Suggests a New Family in the Class Caudoviricetes
Source: Viruses. 2023 Dec 15;15(12):2437. doi: 10.3390/v15122437 (PMC10747016; doi:10.3390/v15122437)
Supplement: Supplementary file 1 [file viruses-15-02437-s001.zip › Supplementary/Data S2.pdf]

No	No CEMTC	Isolation source	Date of isolation	Species identification using 16SrRNA sequencing	GenBank ID
1	1878	animal	2014.05.22	Stenotrophomonas sp. (S.maltophilia/[Pseudomonas] hibiscicola)	MZ424751
2	2142	environmental	2015.04.07	Stenotrophomonas maltophilia	MZ424754
3	1947	environmental	2012.11.26	Stenotrophomonas sp. (S. lactitubi/"S. cyclobalanopsidis")	MZ424758
4	3963	environmental	2020.10.06	Stenotrophomonas sp. (S.maltophilia/[Pseudomonas] hibiscicola)	MZ424759
5	6992	environmental	2022.08.14	Stenotrophomonas maltophilia	OQ353082
6	7417	environmental	2022.08.24	Stenotrophomonas rhizophila	
7	5250	environmental	2021.10.28	Stenotrophomonas lactitubi	OQ913916
8	4548	environmental	2021.10.28	Stenotrophomonas chelatiphaga	OP393921
9	4550	environmental	2021.10.28	Stenotrophomonas sp.	ON838135
**10**	**4565**[1]	**environmental**	**2021.10.28**	**Stenotrophomonas rhizophila**	**OP393922**
11	4860	environmental	2021.10.28	Stenotrophomonas rhizophila	ON838137
12	3863	environmental	2019.10.14	Stenotrophomonas rhizophila	MZ424756
13	3880	environmental	2019.10.24	Stenotrophomonas rhizophila	MZ424757
14	5179	environmental	2021	Stenotrophomonas bentonitica	
15	5206	environmental	2021.11.04	Stenotrophomonas chelatiphaga	OP393923
16	5208	environmental	2021.11.04	Stenotrophomonas rhizophila	OP393924
17	5297	environmental	2022.04.22	Stenotrophomonas chelatiphaga	OP393925
18	5540	environmental	2021.11.10	Stenotrophomonas chelatiphaga	OQ353080
19	6173	environmental	2022.05.11	Stenotrophomonas rhizophila	OQ353081
20	6445	environmental	2021	Stenotrophomonas sp (S. rhizophila/S. bentonitica)	
21	6500	environmental	2022.05.14	Stenotrophomonas rhizophila	
22	6504	environmental	2022.05.14	Stenotrophomonas rhizophila	OP703677
23	6609	environmental	2022.07.17	Stenotrophomonas rhizophila	OP703679
24	7007	environmental	2022.07.21	Stenotrophomonas rhizophila	
25	7607	environmental	2022.05.15	Stenotrophomonas sp. (S. lactitubi/"S. cyclobalanopsidis")	OQ353083
26	7824	environmental	2022.05.14	Stenotrophomonas maltophilia group	
27	8351	environmental	2022.10.22	Stenotrophomonas lactitubi	OQ913918
28	8210	environmental	2022.10.15	Stenotrophomonas lactitubi	OQ913917
29	4523	environmental	2021.10.27	Stenotrophomonas chelatiphaga	OP393920
30	5438	environmental	2022.05.07	Stenotrophomonas sp.	OP393926
31	5471	environmental	2022.05.02	Stenotrophomonas sp.	OP393927
32	5507	environmental	2022.05.01	Stenotrophomonas rhizophila	OQ353079
33	5635	environmental	2022.05.02	Stenotrophomonas rhizophila	OQ834583
34	6565	environmental	2022.07.21	Stenotrophomonas tumulicola	OP703678
35	7619	environmental	2022.05.29	Stenotrophomonas sp. (S. lactitubi)	OQ353084
36	1628	environmental	2013.05.13	Stenotrophomonas acidaminiphila	MZ424752
37	1588	environmental	2013.04.13	Stenotrophomonas pavanii	MZ424753
38	1403	environmental	2012.09.14	Stenotrophomonas rhizophila	MZ424755
39	4286	environmental	2021.08	Stenotrophomonas sp.	OP393919
40	7254	human	2022.09	Stenotrophomonas geniculata	

41	7300	human	2022.09	Stenotrophomonas sp. (S. geniculata/[Pseudomonas] hibiscicola)	OQ353085
42	7260	human	2022.09	Stenotrophomonas geniculata	
43	7315	human	2022.09	Stenotrophomonas geniculata	
44	6838	human	2022.09	Stenotrophomonas sp. (S. geniculata/[Pseudomonas] hibiscicola)	OP703680
45	2164	human	2015.05.26	Stenotrophomonas maltophilia	MZ424760
46	2355	human	2016.01.26	Stenotrophomonas maltophilia	OP393915
47	2356	human	2016.01.26	Stenotrophomonas maltophilia	
48	3806	human	2019.10.23	Stenotrophomonas maltophilia	MZ424764
49	4255	human	2017.07.11	Stenotrophomonas pavanii	OP393916
50	3051	human	2017.10	Stenotrophomonas maltophilia	MZ424761
51	3052	human	2017.10	Stenotrophomonas maltophilia	MZ424761
52	3128	human	2017.10.17	Stenotrophomonas maltophilia	MZ424768
53	2329	human	2016.01.07	Stenotrophomonas maltophilia	MZ424765
54	2517	human	2016.03.29	Stenotrophomonas maltophilia	MZ424766
55	2650	human	2016.07.11	Stenotrophomonas maltophilia	MZ424767
56	4125	human	2021.02.11	Stenotrophomonas maltophilia	MZ424762
57	4225	human	2021.06.24	Stenotrophomonas maltophilia	
58	4227	human	2021.06.24	Stenotrophomonas maltophilia	
59	3453	human	2018.08.21	Stenotrophomonas sp.	
60	3773	human	2019.09.05	Stenotrophomonas maltophilia	MZ424763
61	3659	insect	2019.04.09	Stenotrophomonas maltophilia	MT040043
62	3664	insect	2019.04.09	Stenotrophomonas maltophilia	MT040044
63	3670	insect	2019.04.09	Stenotrophomonas maltophilia	MT040045
64	3672	insect	2019.04.09	Stenotrophomonas maltophilia	MT040046

[1] host strain marked with bold
