# Supplementary figures and images for: A Novel Podophage StenR_269 Suggests a New Family in the Class Caudoviricetes
Source: Viruses. 2023 Dec 15;15(12):2437. doi: 10.3390/v15122437 (PMC10747016; doi:10.3390/v15122437)

SignalP 6.0 prediction: Sequence

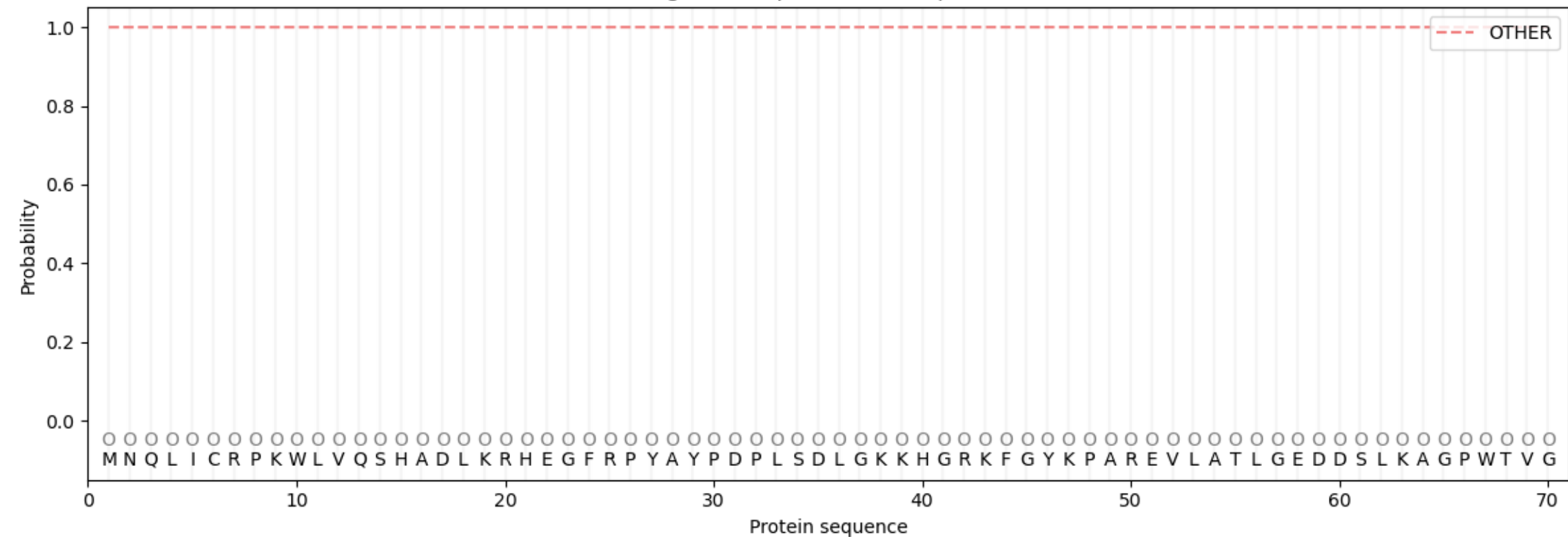

Supplement: Supplementary file 1 [file viruses-15-02437-s001.zip › Supplementary/Data S5.pdf]

Left line: Virus family

Others (15)

Right line: Host group

Pseudomonadota (45)

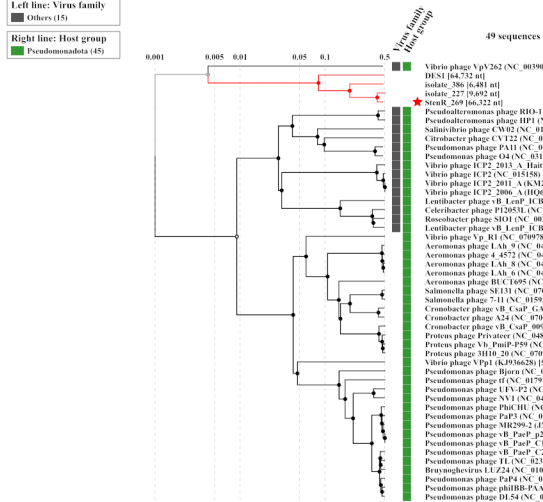

A

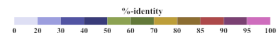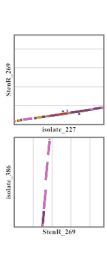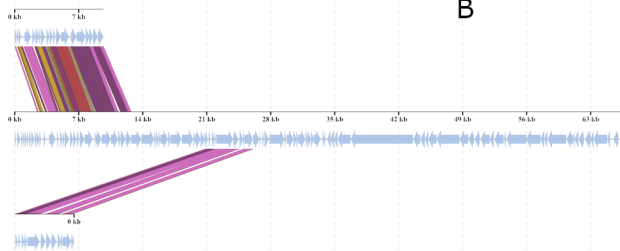

B

Supplement: Supplementary file 1 [file viruses-15-02437-s001.zip › Supplementary/Data S6.pdf]

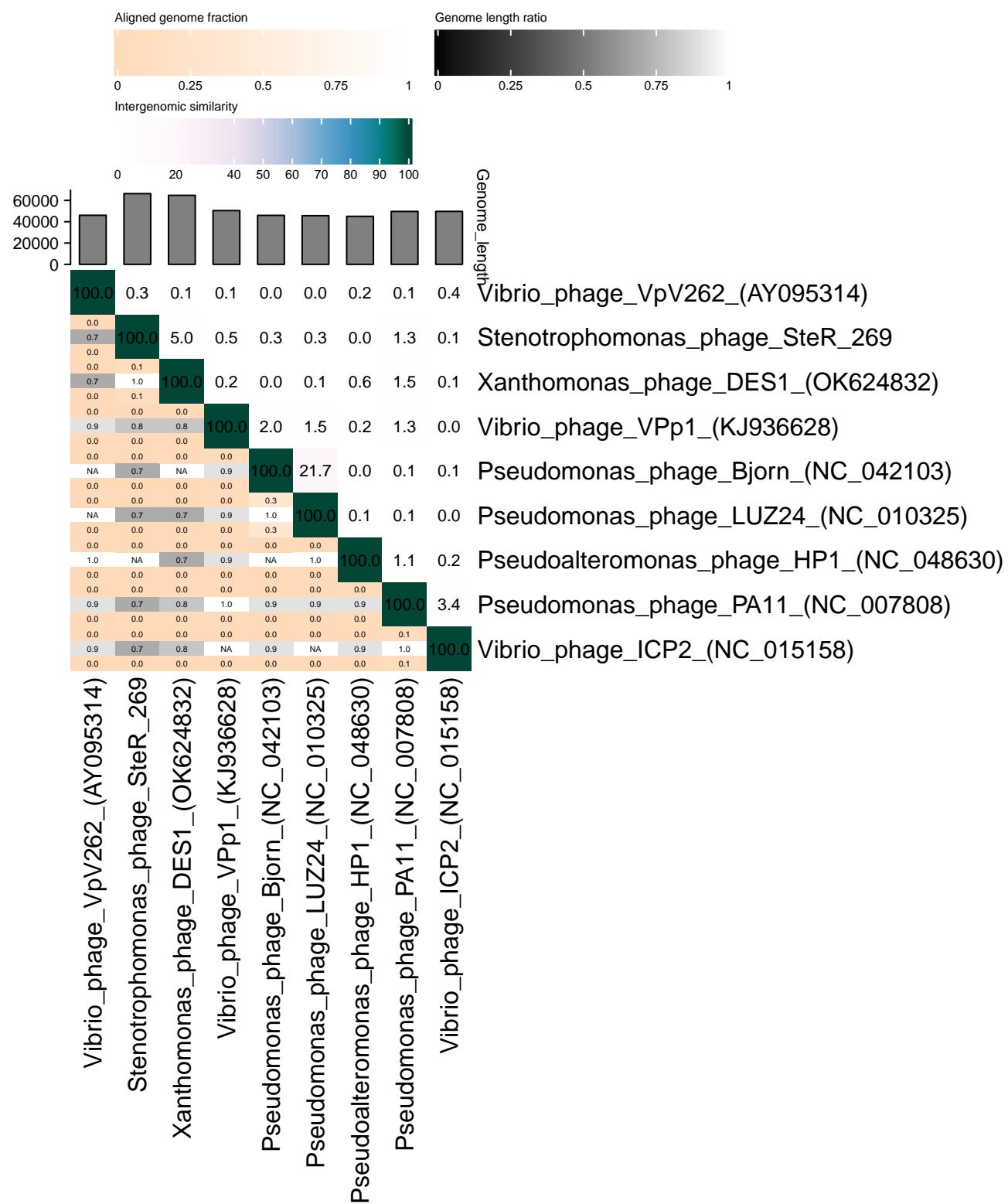

Supplement: Supplementary file 1 [file viruses-15-02437-s001.zip › Supplementary/Data S7.pdf]
